# Apples to apples? Discordant definitions still hinder evidence‐based treatments for sarcopenia

**DOI:** 10.1002/jcsm.13339

**Published:** 2023-09-21

**Authors:** Giulia Coletta, Brad S. Currier, Stuart M. Phillips

**Affiliations:** ^1^ Department of Kinesiology, Faculty of Science McMaster University Hamilton Canada

Preservation of skeletal muscle strength, size and function is critical to healthy aging. The progressive loss of each with advancing age—sarcopenia—is deleterious on several levels. Exercise is the primary countermeasure for sarcopenia, but ‘exercise’ alone is a broad recommendation. In a recent publication in the Journal of Cachexia, Sarcopenia and Muscle, Shen and colleagues^1^ aimed to compare the effectiveness of different exercise interventions for improving relevant patient outcomes (e.g., all‐cause mortality, quality of life and falls) in older adults with sarcopenia. The authors summarized the effect of 12 interventions from 42 randomized controlled trials using a network meta‐analysis (NMA). Resistance training (strength training) was the most effective exercise for improving quality of life, strength and physical performance, with or without additional exercise/nutritional interventions. The authors^1^ deserve substantial recognition for their comprehensive work. In our view, their results support and reinforce resistance training as the primary countermeasure to sarcopenia^2^ but also exemplify a major issue: disparate definitions impede the diagnosis and evidence‐based treatment of sarcopenia.

Resistance training is an effective intervention to combat age‐related non‐communicable disease, co‐morbidities and loss of physical function, all of which impact older adults' ability to accomplish activities of daily living (e.g., feeding, bathing, dressing and toileting).^3^ All physical activity and exercise types benefit older adults; however, resistance training is most effective at improving muscle strength and power, augmenting (or mitigating loss of) muscle mass and improving physical function, all of which help maintain independence and improve their overall quality of life as they age.^4^ Consequently, experts have emphasized resistance training as a first‐line strategy to prevent and manage sarcopenia.^2^, ^5^ Recent evidence suggests that resistance training programmes consisting of two exercise sessions per week (upper‐ and lower‐body exercises) performed with a relatively high degree of effort are appropriate for treating sarcopenia.^6^ Despite the benefits, exercise is not integrated as a standard component of care in geriatric medicine, and a call to action for personalized exercise prescriptions tailored to older adults is needed.^7^ Shen and colleagues^1^ provided foundational evidence that resistance training should be considered before or with other treatments for sarcopenia—however, the longstanding lack of a consensus definition for sarcopenia limits research and clinical application.

Recently, network meta‐analysis has climbed to the forefront of evidence‐based medicine. Compared to pairwise meta‐analyses, NMA utilizes direct and indirect evidence to simultaneously compare, rank order and calculate the effect of multiple interventions.^8^ A key NMA assumption is transitivity: All included trials are jointly randomizable.^9^ In other words, the participants in each included study should be eligible for all other studies in the NMA to reduce confounding variables when calculating the effect of interventions within a population. For example, if someone wishes to determine the best baking temperature for an apple pie, they could compare different baking temperatures (intervention) and the resulting apple pies (outcome). However, the true effect of baking temperature could not be determined if distinct apple types (populations) were not differentiable and various apple types were used in the comparisons. Extend our apple pie example to comparisons of exercise interventions on functional outcomes for individuals with ‘sarcopenia’ who are simply different apples. The definition of sarcopenia is inconsistent and disputed,^10^ so the precise effect—and ranking—of exercise interventions on individuals with sarcopenia cannot be adequately determined.

The criteria ‘accepting the authors [of included studies] definition of sarcopenia’ or simply stating ‘individuals with sarcopenia’ are often used in evidence synthesis both introduce the risk of including heterogeneous populations and potentially violating transitivity assumptions and likely lower the precision of the effect estimate. Two alternative solutions to the sarcopenia definition dilemma are including all adults over a predefined biological age^11^ or employing and specifying an existing sarcopenia framework. However, both solutions lack precision for sarcopenia‐specific treatments and involve notable limitations.^10^ Thus, a unified consensus definition of sarcopenia is needed to develop evidence‐based treatments.

The definition of sarcopenia continues to evolve, creating difficulty in determining the diagnosis and prognosis of the newly classified disease. The most common definitions include a combination of (a) muscle mass [measured using proxies of muscle mass—appendicular lean soft tissue via dual‐energy X‐ray absorptiometry (DXA) or bioelectrical impedance analysis (BIA)]; (b) muscle strength (often measured using hand grip strength); and (c) physical function (measured using gait speed). However, each consensus definition uses different combinations of muscle mass, strength and physical function to operationalize the definition of sarcopenia. Additionally, each group recommends various measures and cutoff points to capture these outcomes.^10^ For example, the European Working Group on Sarcopenia in Older People (EWGSOP) recommends appendicular lean mass for muscle mass, grip strength or chair stand for muscle strength, and gait speed, short performance physical battery, timed up and go, or 400‐m walk test for physical function. The Asian Working Group for Sarcopenia (AWGS) uses DXA or BIA, grip strength and 6‐m gait speed for muscle, strength and function, respectively. Using different consensus group definitions results in differences in the prevalence of sarcopenia, and there would be, at best, a modest agreement between the various definitions,^12^ which would be attributed to the lack of criterion standards.

Standardized protocols to define sarcopenia would enable better comparison of research outcomes across studies and facilitate the identification of optimal resistance training methods to counteract muscle loss and improve physical function in individuals with sarcopenia. Collaboration between researchers, healthcare providers and professional organizations is essential to achieve consensus on resistance training prescriptions for sarcopenia. In August 2021, the Global Leadership Initiative in Sarcopenia (GLIS) working group was formed by members of the Australian and New Zealand Society for Sarcopenia and Frailty Research, the AWGS, the EWGSOP and Sarcopenia Definitions and Outcome Consortium. The primary goal of the GLIS is to produce an inclusive definition of sarcopenia that can be utilized globally.^13^

Shen and colleagues^1^ should be commended for their valuable contribution to establishing clinical practice guidelines on optimal exercise interventions for older individuals with sarcopenia. The absence of a consensus definition for sarcopenia continues, however, to hinder the development and prescription of treatments for the disease.^10^ Future work needs to focus on developing a true consensus definition for sarcopenia, as without it, recommendations for its treatment are rudderless.
